# EEG Microstates Change in Response to Increase in Dopaminergic Stimulation in Typical Parkinson’s Disease Patients

**DOI:** 10.3389/fnins.2018.00714

**Published:** 2018-10-15

**Authors:** J. Ignacio Serrano, María Dolores del Castillo, Verónica Cortés, Nuno Mendes, Aida Arroyo, Jorge Andreo, Eduardo Rocon, María del Valle, Jaime Herreros, Juan Pablo Romero

**Affiliations:** ^1^Neural and Cognitive Engineering Group, Centre for Automation and Robotics, Spanish National Research Council – Technical University of Madrid, Madrid, Spain; ^2^Faculty of Experimental Sciences, Francisco de Vitoria University, Madrid, Spain; ^3^Faculty of Sciences, University of Lisbon, Lisbon, Portugal; ^4^Department of Neurology, Fuenlabrada University Hospital, Madrid, Spain; ^5^Department of Neurology, Infanta Leonor University Hospital, Madrid, Spain; ^6^Brain Damage Unit, Hospital Beata Maria Ana, Madrid, Spain

**Keywords:** Parkinson’s disease, electroencephalography, microstates, levodopa, diagnosis

## Abstract

**Objectives:** Characterizing pharmacological response in Parkinson’s Disease (PD) patients may be a challenge in early stages but gives valuable clues for diagnosis. Neurotropic drugs may modulate Electroencephalography (EEG) microstates (MS). We investigated EEG-MS default-mode network changes in response to dopaminergic stimulation in PD.

**Methods:** Fourteen PD subjects in HY stage III or less were included, and twenty-one healthy controls. All patients were receiving dopaminergic stimulation with levodopa or dopaminergic agonists. Resting EEG activity was recorded before the first daily PD medication dose and 1 h after drug intake resting EEG activity was again recorded. Time and frequency variables for each MS were calculated.

**Results:** Parkinson’s disease subjects MS A duration decreases after levodopa intake, MS B appears more often than before levodopa intake. MS E was not present, but MS G was. There were no significant differences between control subjects and patients after medication intake.

**Conclusion:** Clinical response to dopaminergic drugs in PD is characterized by clear changes in MS profile.

**Significance:** This work demonstrates that there are clear EEG MS markers of PD dopaminergic stimulation state. The characterization of the disease and its response to dopaminergic medication may be of help for early therapeutic diagnosis.

## Introduction

Parkinson’s Disease (PD) is a neurodegenerative disease affecting up to 3% of the population ≥ 65 years of age (Poewe et al., 2017). PD has been associated with several risk factors common to other age related diseases and some chemicals exposure (Beitz, 2014) but its ultimate cause is still unknown.

In typical PD, progressive degeneration of dopaminergic neurons in the substantia nigra is correlated with the wide known motor symptoms of bradykinesia, rigidity, and tremor (Kalia and Lang, 2015). However, the phenotypical profile of each patient gives rise to the identification of several subtypes such as tremor-dominant subtype and on the other hand bradykinesia/rigidity dominant (Marras and Lang, 2013). Dopaminergic treatment usually provides substantial alleviation in motor symptoms. Some other symptoms such as gait disturbance and postural instability do not usually have a substantial improvement (Hely et al., 2005). There are several syndromes that share some features similar to Parkinson’s disease, but the progression and onset of symptoms are different. One of the main characteristics of this atypical Parkinsonism is the lack of or incomplete response to levodopa (Goetz et al., 2008; Stamelou and Hoeglinger, 2013).

Levodopa is actually considered the best current symptomatic treatment for Parkinson’s Disease. One of the main obstacles for the treatment of the disease is its pharmacodynamics, meaning there is a low penetrance of the drug into the central nervous system (Khor and Hsu, 2007; Britz et al., 2010; Musso et al., 2010; Van de Ville et al., 2010; Michel and Koenig, 2017). The action of this drug determines important changes not only in motor but also in non-motor symptoms. The state where patients show a marked improvement is called “ON state” and the one with no effects is called “OFF state.” Dopamine (DA) receptor agonists are also used to treat the symptoms of the disease since such drugs mimic the action of dopamine, their action is achieved by stimulation of pre-synaptic (auto receptors) and post-synaptic DA receptors (Radad et al., 2005). Levodopa equivalent dose (LED) can be calculated from dopamine receptor agonist doses so the total daily levodopa administration can be estimated (Tomlinson et al., 2010).

Dopaminergic stimulation is surely alleviating typical PD symptoms in most patients, but the degree of its effects shows major inter-individual differences. These differences in levodopa motor response are evident even between same disease-severity-stage patients (Goetz et al., 2000). Every patient has their own needs of medication to reach their “ON state” and this varies according to the disease progression (Nyholm et al., 2012) and degree of denervation (Kostrzewa et al., 2005).

The clinical diagnosis of PD is currently based on clinical symptoms and other support criteria such as response to medication. This clinical diagnosis is very difficult, especially in early stages of the disease when there are no remarkable motor features.

Identification of neurophysiological variables with diagnostic value in early-stage PD would raise the chances of improving diagnostic certainty (Valls-Solé and Valldeoriola, 2002).

Electroencephalography (EEG) is a well-known technique used to record the electrical field produced by the electrical activity in the brain. This technique is characterized by a high temporal resolution and high test–retest reliability (Lopes da Silva, 1991). It has been published that quantifying EEG rhythms and their variations could be the source of biomarker for several neuropsychiatric disorders, such as schizophrenia, major depressive disorder, or even neurodegenerative diseases as Alzheimer’s disease (Gandal et al., 2012; Han et al., 2013).

Electroencephalography data can be analyzed according to momentary states of the topographical brain activation, called microstates (MS). “Microstate analysis is a method in which states are defined by topographies of electrical potentials on a set of multichannel electrodes that remain stable for 80–120 ms before rapidly moving to a different microstate” (Khanna et al., 2015). Unlike other EEG processing techniques, in microstates, the simultaneous analysis of the signals from all the electrodes is used to create a global representation of a functional state. In fact, many studies have shown that time series of EEG microstates vary through behavioral states (Stevens and Kircher, 1998; Lehmann et al., 2010), personality types (Schlegel et al., 2012) and neuropsychiatric disorders (Dierks et al., 1997; Lehmann et al., 2005; Kikuchi et al., 2011; Khanna et al., 2015). Consequently, changes in the duration or frequency of appearance of specific microstates can be considered as biomarkers for different neurological and neuropsychiatric conditions.

Interestingly, several studies that have examined resting-state EEG report the same four archetypal microstates that explain most of the global topographic variance (Koenig et al., 2002; Khanna et al., 2015). The four canonical EEG microstates (A, B, C, D) seem to represent the neurophysiological correlates of four known Resting State Networks identified by fMRI, suggesting that Resting State Networks of fMRI may be the same ones that give rise to microstates (Michel and Koenig, 2017). The dynamics of these networks may imply various brain functions, and their alteration can be associated with the pathophysiology of several neurological and neuropsychiatric diseases (Khanna et al., 2015).

When the microstate time series is convolved with the resting-state fMRI BOLD signal, each microstate map correlates with the activity of particular RSNs (Britz et al., 2010; Musso et al., 2010; Yuan et al., 2016). Britz et al. (2010) showed that microstate A is associated with the phonological processing network, B with the visual network, C with the salience network, and D related to the attentional network.

There is evidence that neurotropic drugs may modulate EEG microstates (Kikuchi et al., 2007, 2011; Yoshimura et al., 2007), but there are no studies showing EEG microstate changes in response to the administration of levodopa or dopaminergic agonist drugs in typical PD patients.

The aim of the study presented in this paper is to identify EEG microstate changes that characterize levodopa response. The data obtained from this study can be used to support typical PD diagnosis in difficult clinical scenarios where a therapeutic trial of levodopa is not feasible or not well tolerated by the patient because of the gastric effects of its administration.

## Materials and Methods

### Participants

The protocol was approved by the CEIC Fuenlabrada Hospital, Madrid, Spain. All subjects gave written informed consent in accordance with the Declaration of Helsinki.

Fourteen patients were included in the study after signing informed consent forms (4 female: mean age 66.25 ± 12.9, range 52–80 and 10 male: mean age 66.9 ± 7.41, range 50–76). All the patients had been diagnosed with Parkinson’s Disease according to London Brain Bank criteria (mean time from onset 7.29 ± 2.33 years, range 4–13), with Hoehn and Yahr scale 2 ± 0.8 (range 1–3), and were taking levodopa or dopaminergic agonists (mean daily amount 324.28 ± 232.77 levodopa milligrams equivalent dose (LED), range 100–733) in stable dosing regimen for at least 90 days with a clear ON effect (good clinical effect). There was not head tremor in any of the patients. **Table 1** shows a description of included patients. In addition, twenty-one healthy subjects were recruited as control participants (6 female: mean age 67.4 ± 10.21, range 50–77 and 15 male: mean age 69.6 ± 10.14, range 50–93).

**Table 1:** Description of the patients included in the study.

	Gender	Age	Initial side	Years from onset	Hoehn Yahr	UPDRS^∗^	Levodopa equivalent dose (mg) (LED)	Levodopa dose (mg)	Total morning dose (mg)
1	Male	73	Right	8	1	14	100^2,3^	50	150
2	Male	67	Left	6	1	8	100^2^	0	100
3	Female	59	Left	6	1	10	420^2,4^	50	470
4	Female	74	Left	9	3	12	400^2,5^	250	650
5	Male	71	Left	8	2.5	15	0	100	100
6	Female	80	Right	9	3	14	520^2,4,7^	200	720
7	Male	76	Left	13	3	30	220^3,5,6^	0	220
8	Male	50	Left	5	2	24	100^2^	0	100
9	Male	68	Left	6	2	11	310^1,2^	0	310
10	Male	69	Left	6	1	22	105^1^	250	355
11	Male	69	Left	8	2	16	205^1,2,3^	100	305
12	Female	52	Right	4	2	12	100^2^	67	167
13	Male	59	Right	9	3	11	550^1,3,5,6^	283	833
14	Male	67	Right	5	1.5	11	260^2,4^	0	260
Avg (std)	28.57% Female	66.71 (8.77)	35.71% Right	7.29 (2.33)	2.00 (0.81)	14.46 (6.09)	504.00 (366.68)	96.43 (105.39)	338.57 (242.17)

*^∗^UPDRS, Unified Parkinson’s Disease Rating Scale. ^1^Pramipexole, ^2^Rasagiline, ^3^Benserazide, ^4^Ropirinole, ^5^Rotigotine, ^6^Safinamide, ^7^Amantadine.*

### Intervention

**FIGURE 1:**
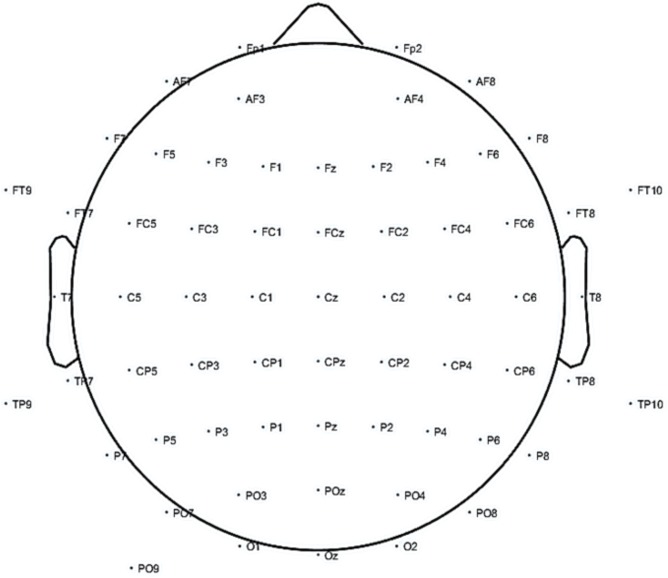
Electroencephalography (EEG) electrode placement for the study.

Participants were asked to come to the hospital early in the morning without their corresponding daily levodopa or agonist intake (at least 8 h after the last levodopa or dopamine agonist dose). Resting EEG activity was recorded over 2 min by 64 electrodes placed according to the 10–20 system as depicted in **Figure 1**. They were comfortably seated with their hands on their laps, relaxed jaw and eyes open, looking at a white wall. Immediately afterward, the EEG electrodes were removed, and they took their daily Levodopa or agonist dose with a glass of water, 30 min before they had a light breakfast and were given free time. The resting EEG activity was analogously recorded 1 h after the levodopa intake, once the patient had asserted they were in their usual ON state.

### Materials

An actiCHamp amplifier (Brain Vision LLC, NC, United States) was used to amplify and digitize the EEG data at a sampling frequency of 512 Hz. The EEG data were stored in a PC running Windows 7 (Microsoft Corporation, Washington, DC, United States). EEG activity was recorded from 64 positions with active Ag/AgCl scalp electrodes (actiCAP electrodes, Brain Vision LLC, NC, United States). The ground and reference electrodes were placed on AFz and on FCz, respectively (see **Figure 1**).

Electroencephalography acquisition was carried out by NeuroRT Studio software (Mensia Technologies SA, Paris, France). The EEG signal processing procedure was performed using MATLAB functions (MathWorks Inc., Natick MA, United States), specifically the EEGLab toolbox (Delorme and Makeig, 2004). EEG microstates were extracted and characterized by LORETA-KEY v20170220 software (the Key Institute for Brain-Mind Research, Zurich, Switzerland). Statistical analyses were performed by SPSS for Windows, version 23.0 (IBM Inc., Chicago, IL, United States).

### EEG Processing and Outcome Measurements

The continuous EEG signal for each channel was artifact-corrected by the Artifact Subspace Reconstruction algorithm (Mullen et al., 2013), disabling all parameters except the high-pass filter band width (0.25–0.75) and burst repairing (*kurtosis* > 5). The signal was then band-pass filtered between 2 and 31 Hz with a Finite Impulse Response (FIR) filter (order 846). Finally, a common average reference (CAR) spatial filter was applied.

The processed EEG was the input to an EEG microstate detection and characterization algorithm (Pascual-Marqui et al., 2014). The algorithm requires an initial interval for the number of microstates searched. This interval was set to 4–10. The algorithm was run independently for PD PRE, PD POST and CONTROL conditions. Mean microstate topographies in each condition were manually assigned to canonical microstates reported in previous studies. The assignment was individually performed by three judges (among the authors) by visual analysis. This procedure avoids the likely mislabeling introduced by the common topography correlation analysis in the presence of irregular topographies and more than four microstates (Custo et al., 2017). Labels agreed by two or more judges were assigned (all mean microstate topographies labels were agreed by two judges at least). From this algorithm, the microstates accounting for most of the variance were selected. For each microstate, the percentage of the total time in the microstate (coverage), the percentage of times entered in the microstate (frequency), the number of times entered in the microstate (occurrence) and the average duration of the microstate are calculated. In addition, the frequency and probability of change from each microstate to each other one are also calculated, giving a total of 6 features for each microstate.

### Statistical Analysis

The microstate features mentioned above are compared between the pre- and post-levodopa intake conditions. The difference of averages between pre- and post-conditions for each feature was checked by a *t*-test for repeated measurements with bootstrapping (*n* = 1000). Differences with a significance *p* < 0.05 and confidence intervals (lower and upper) with the same sign were considered as statistically significant.

## Results

**FIGURE 2:**
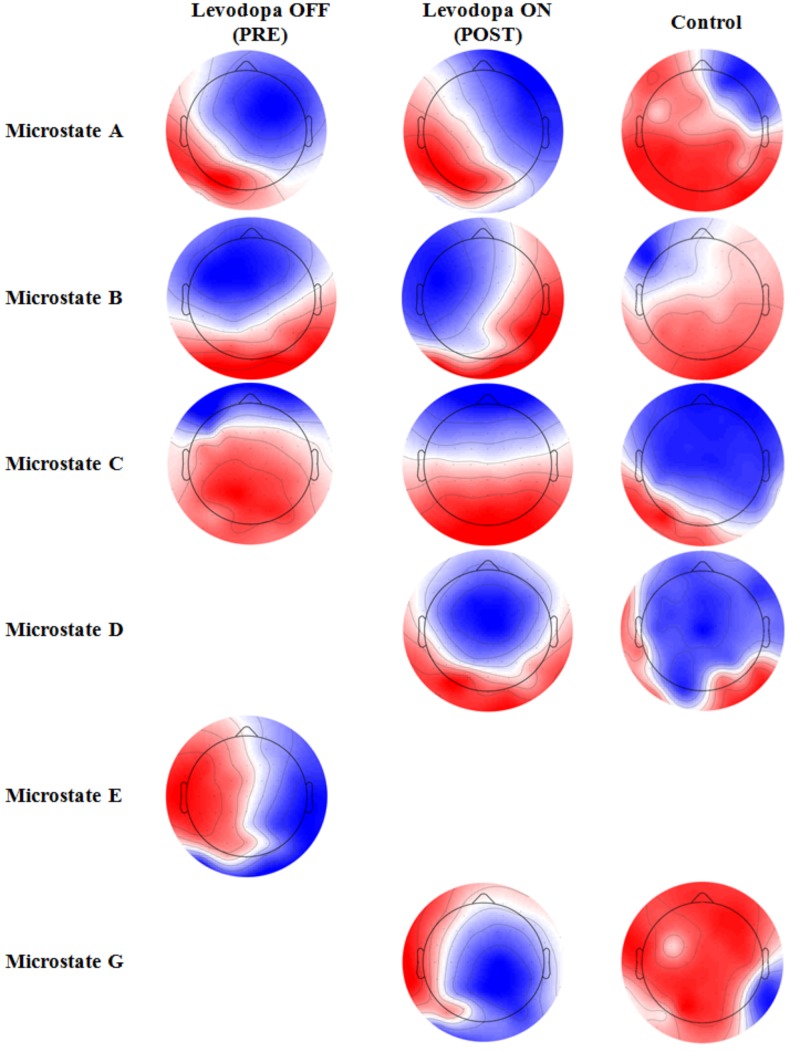
Average topographic distributions of found microstates in pre- and post-levodopa intake conditions and control condition.

**Figure 2** shows the microstates topographies found in pre- (first column) and post- (second column) levodopa intake conditions. Control group (third column) is examined with no medication.

In the pre-condition (OFF state), canonical A, B and C microstates were found with a percentage of explained variance of 19.55, 20.34 and 18.55%, respectively. Canonical microstate D was not identified. Microstates B and C presented altered patterns with respect to the findings in the literature (Michel and Koenig, 2017). In the absence of levodopa intake, a microstate E was also found, in congruence with the definition of Custo et al. (2017), with an explained variance of 19.58%.

After levodopa intake (ON state), the four canonical microstates A, B, C, and D were found with normal patterns (Michel and Koenig, 2017), and explained variances of 20.12, 18.39, 20.00, and 16.67%, respectively. Microstate E was no longer present after levodopa intake. However, the microstate G, according to Custo et al. (2017), was found with an explained variance of 15.39%. Control participants presented the same latter microstates (**Figure 2**, third column), with A, B, C, D, and G percentages of global explained variance of 20.85, 22.51, 17.49, 7.06, and 12.42%, respectively.

Given that the two patient conditions only share three microstates (A, B, and C), **Table 2** presents the statistically significant differences in the features of those three microstates between the OFF and ON states. The remaining features not present in **Table 2** did not show significant differences.

**Table 2:** Statistically significant differences in features of Electroencephalography (EEG) microstates between pre- and post-levodopa intake in Parkinson’s patients.

*N* = 14	*Average difference (POST-PRE)*	*Bootstrapping simulation*			
		*SE*	*p*	*95% CI*	
				*Lower*	*Upper*
Average duration A	−0.00006864	0.00002078	0.009	−0.00010	−0.000031
Occurrence B	549.28571	47.85855	< 0.0001	419.67657	642.10915
Frequency B to A	−680.17248	214.30544	0.033	−1026.77105	−271.71723

*SE, Standard error; p, significance value; CI, Confidence interval.*

According to the results in **Table 2**, the microstate A shows a decreased duration after levodopa intake. Moreover, microstate B appears more often than before levodopa intake. Finally, the frequency of transition from microstate B to A got decreased with levodopa.

**Table 3** presents the statistically significant differences in microstate features between control participants and patients after levodopa intake. No significant differences in microstate features were found between control participants and patients before levodopa intake. All microstate types except B occurred more often in post-levodopa patients than in control participants. This difference is especially high for microstate D, which also presented a higher explained variance and coverage. The probability of shifting from all microstates to D was also higher in post-levodopa patients, as is justified by the mentioned increased occurrence of the latter.

**Table 3:** Statistically significant differences in features of EEG microstates between post-levodopa intake of Parkinson’s patients and control participants.

*N* = 21	*Average difference (Control-PD POST)*	*Bootstrapping simulation (n = 1000)*			
		*SE*	*p*	*95% CI*	
				*Lower*	*Upper*
% EV D	−5.198024	1.425808	0.004	−8.223715	−2.748249
Coverage D	−12.136262	2.076990	0.001	−16.321933	−8.071208
Average duration D	−0.00009076	0.00003172	0.024	−0.00015279	−0.00002818
Occurrence A	−86.500	33.004	0.015	−150.628	−21.874
Occurrence C	−89.167	31.995	0.011	−154.717	−28.991
Occurrence D	−213.238	26.329	0.001	−262.567	−161.391
Occurrence G	−106.167	34.890	0.005	−171.316	−37.608
Probability A to D	−0.15608055	0.025070321	0.001	−0.207682815	−0.10978173
Probability B to D	−0.134840881	0.021954275	0.001	−0.175374815	−0.0919498
Probability C to D	−0.174758333	0.026128700	0.001	−0.229032260	−0.1263252
Probability G to D	−0.111732048	0.027221222	0.001	−0.163486738	−0.0606472

*EV, Explained variance; SE, Standard error; p, significance value; CI, Confidence interval.*

## Discussion

The fact that control subjects and PD patients after taking levodopa show the same microstate types is supported by the fact that levodopa is known to restore altered motor and non-motor functions in PD patients.

A higher duration of microstate A has been related to clinical variables such as disability and cognitive fatigue in patients with multiple sclerosis (Gschwind et al., 2016), and could be related to the cognitive fatigue presented by patients with PD. Such fatigue decreases with dopaminergic stimulation, and therefore it can lead to a decrease in the duration of microstate A, as observed in the results obtained in our study. As we said earlier that Britz et al. (2010) showed that microstate B is associated with the visual network, then the observed increase in the number of times microstate B is present after levodopa or dopaminergic agonist intake might be explained by a lower fatigability in visual monitoring and a better functioning of PD patients thanks to the medication (Lou, 2009, 2015), reflecting the tendency of visual network generators to be active in the absence of cognitive fatigue (Milz et al., 2017). Cognitive fatigability is most likely associated with neurotransmitter (dopaminergic, cholinergic, and noradrenergic) abnormalities in PD. Levodopa may be effective in treating fatigue and fatigability (Lou, 2015). The decrease of frequency of transition from microstate B to A with dopaminergic stimulation does not seem to be related to any known clinical or behavioral condition. Therefore, more research is needed on the importance and functional correlation of the transition of microstates.

There are no differences in characteristics for the microstates shared by patients before medication intake and the controls. However, after levodopa intake there are differences, mainly in the microstate D. There are studies that demonstrate reduced duration (Kikuchi et al., 2007; Nishida et al., 2013) and also a lower frequency of appearance (Lehmann et al., 2010) of microstate D in patients with schizophrenia. Schizophrenia is believed to have a dopaminergic deficit up to a certain point that could explain this common finding in PD (Van Den Brink et al., 2018). Consequently, it is to be expected that patients diagnosed with PD have a lower frequency of appearance of microstate D before taking the medication. Patients presented a greater frequency and duration of microstate D after increasing dopaminergic stimulation, as a consequence of taking the medication. This increase is even greater than in healthy controls, which, assuming that microstate D reflects dopaminergic activity, could be a result of an acute increase of this activity in the brain.

Regarding topographic considerations, microstate D is mainly due to the activation of the right inferior parietal (BA40), the right middle and superior frontal gyri and the right insula (BA13) (Custo et al., 2017). The right inferior parietal area is related to executive control and vision-guided movements (Lasaponara et al., 2018) and the insula has a direct relationship with motor planning (Beurze et al., 2007). The right middle and superior frontal areas seem to explain the changes related to the improvement in attention (Angelidis et al., 2018). The appearance of microstate D, after taking medication, is congruent and consistent with the disappearance of certain motor and non-motor symptoms after levodopa.

Apart from the four canonical microstates (A–D) (Custo et al., 2017), two additional microstates (E and G) were identified. The microstate E corresponds to the activation of the middle frontal gyrus, the dorsal part of the anterior cingulate, the cuneus and the thalamus. Dopamine has an inhibitory effect (D2 receptors). Consequently, it is plausible to attribute a relative hyperactivity in its absence to the thalamus, and that this relative hyperactivity disappears after the intake of levodopa. Besides, according to Yoo et al. (2015), the anterior cingulate and frontal areas correspond to the presence of non-motor symptoms. Therefore, this justifies the presence of microstate E in patients before dopaminergic stimulation and its disappearance after they took levodopa (Lou, 2009, 2015). Finally, the cuneus is also related with oculomotor control (Darby et al., 1996), which is well-known to also be a function specifically regulated by the basal ganglia, whose function is altered in PD (Pretegiani and Optican, 2017).

The microstate G corresponds to the activation of the right inferior parietal lobe, extending to the superior temporal gyrus and also the cerebellum (Khanna et al., 2015). Both areas are closely related to motor behavior (Pirondini et al., 2017). Therefore, the appearance of microstate G after levodopa intake is strongly consistent with the improvement of motor symptoms. Since the visible and clinically evaluable motor symptoms disappear with the medication intake, this results in the observation of the microstate G.

The present study is not without limitations. First, the sample size is relatively small. A larger population might have yielded more significant results. Second, the sample population is heterogeneous in medication terms, with different types and doses of drugs, although they are all in their optimal ON state. Heterogeneity in medication is usual in PD patient cohorts because such variations correspond to the different treatment strategies that can be initiated even in the same disease stage. Dopaminergic agonists play a key role in actual treatment of the disease and their diversity makes them comparable only by their conversion to dopaminergic equivalents as we did in our study. Besides, cognitive fatigue, related to microstates A and B, was not assessed in this study. Cognitive fatigability and cognitive fatigue are usually evaluated through self-reporting scales. Given that our measures were performed in sequential OFF and ON state (in less than 2 h), we considered that this evaluation would have had a very important bias of motor and emotional symptoms as product of dopaminergic deprivation. In addition, dopaminergic therapy optimization is one of the main management recommendations for treating fatigue in PD (Kostić, 2016). Finally, no cognitive evaluation was performed, specially attention changes that are highly related to microstate D. Nevertheless, cognitive symptoms improvement, included attention span, has been widely reported in PD patients in response to dopaminergic treatment and can be assumed as a well stablished effect of medication. These limitations should be taken into consideration when interpreting the conclusions of the study.

## Conclusion

Electroencephalography microstate analysis can be performed by means of an economical and minimally invasive technique with high temporal resolution. Since the EEG microstate correlation with RSNs has been evidenced, the results obtained from microstate analysis have been interpreted based on the known findings about these networks. Our work has demonstrated that there is an alteration of EEG microstate features and occurrence in typical PD in response to levodopa administration. These changes correlate with known clinical effects of the substance on such patients and are coherent with related changes in RSNs.

In spite of the differences between controls and PD patients, the microstates found in patients after levodopa intake are closer to controls’ microstates than before taking the medication. Thus, the analysis performed in this study can be considered as a means to assess the suitability of the patients’ medication dosage.

Further, not every Parkinsonian patient has a good response to levodopa or dopamine agonist treatment, and patients who were non-respondent to levodopa are excluded from typical PD diagnosis. Absence of complete clinical response to Levodopa is common on atypical parkinsonian patients. This lack of effect of dopaminergic stimulation is considered a red flag and implies the exclusion for typical Parkinson’s Disease diagnosis (Postuma et al., 2015). Considering our results, we would not expect a microstate “normalization” in atypical PD patients in response to dopamine administration. Consequently, the microstate analysis can be considered of great utility to characterize the levodopa response prior to making a diagnosis of typical vs. atypical Parkinsonism in a non-invasive way suitable for outpatients. Nevertheless, further studies are required to characterize EEG microstate changes due to levodopa administration on atypical PD patients.

## Author Contributions

VC, JS, MDC, and JR contributed conception and design of the study. AA, JA, NM, JH, MDV, and JR organized the database. JS, MDC, and JR performed the statistical analysis. VC and JR wrote the first draft of the manuscript. VC, JS, MDC, ER, and JR wrote sections of the manuscript. All authors contributed to manuscript revision, read and approved the submitted version.

## Conflict of Interest Statement

The authors declare that the research was conducted in the absence of any commercial or financial relationships that could be construed as a potential conflict of interest.
